# Treatment outcomes in metastatic and localized high-grade salivary gland cancer: high chance of cure with surgery and post-operative radiation in T1–2 N0 high-grade salivary gland cancer

**DOI:** 10.1186/s12885-018-4578-0

**Published:** 2018-06-20

**Authors:** Jeon Yeob Jang, Nayeon Choi, Young-Hyeh Ko, Man Ki Chung, Young-Ik Son, Chung-Hwan Baek, Kwan-Hyuck Baek, Han-Sin Jeong

**Affiliations:** 1 Department of Otolaryngology, Ajou University School of Medicine, Suwon, Republic of Korea; 2 Department of Otorhinolaryngology - Head and Neck Surgery, Samsung Medical Center, Sungkyunkwan University School of Medicine, 81 Irwon-ro, Gangnam-gu, Seoul, 06351 Republic of Korea; 3 Department of Pathology, Samsung Medical Center, Sungkyunkwan University School of Medicine, Seoul, Republic of Korea; 4 Department of Molecular and Cellular Biology, Samsung Biomedical Research Institute, Sungkyunkwan University School of Medicine, Suwon, Republic of Korea

**Keywords:** Salivary gland neoplasm, High-grade pathology, Treatment outcomes, Prognosis

## Abstract

**Background:**

High-grade salivary gland cancer is a distinct clinical entity that has aggressive disease progression and early systemic spread. However, because of the rarity of the disease, the clinical outcomes, prognostic factors and clinical decision on the optimal treatments have not been fully understood.

**Methods:**

In this study, we retrospectively analyzed the clinical data of 124 patients with high-grade salivary gland cancers and performed multivariate survival analyses to evaluate the clinico-pathological factors affecting the treatment outcomes.

**Results:**

The 5-year disease-specific survival was 63.4% in patients with high-grade salivary gland cancers. Among the clinico-pathological factors, presence of lymph node metastasis (hazard ratio 5.63, 95% confidence interval 2.64–12.03, *P* < 0.001) and distant metastasis (hazard ratio 4.59, 95% confidence interval 2.10–10.04, *P* < 0.001) at diagnosis were the most potent unfavorable prognostic factors. Importantly, patients with early-stage disease (T1–2N0M0) showed apparently a relatively excellent prognosis (93.2% 5-year disease-specific survival); meanwhile N (+) and M1 status at diagnosis resulted in dismal outcomes (44.6 and 21.1% 5-year disease-specific survival, respectively). On comparing surgery alone as a treatment modality, surgery plus postoperative radiation significantly benefited the patients, but the difference between adjuvant radiation and chemoradiation was not found to be significant. Pathological subtypes of high-grade salivary gland cancers were not significantly associated with prognosis.

**Conclusions:**

Despite of an overall unfavorable prognosis in high-grade salivary gland cancer, patients with early-stage disease are expected to have excellent prognosis (over 90% survival rates) with surgery plus adjuvant radiation, which may implicate the patients’ consultation, therapeutic decision making, and the need for early detection of the disease.

## Background

Salivary gland cancer is rare (0.6–1.4 per 100,000) and arises from the major and minor salivary glands, and it also has diverse histopathology comprising 21–22 subtypes [1–4]. Diagnosis, estimation of prognosis and decision on the optimal treatments of salivary gland cancer need to be improved because of the rarity of this disease and pathological diversity [5]. Previously, we and other researchers have reported that the critical decision making in the diagnosis and treatment of salivary gland cancer should be based on the histological grade of tumors, and not on the specific pathological subtype [6–8].

Low-grade salivary gland cancers have excellent outcomes even after marginal excision of tumors, similar to those for benign salivary gland tumors [9, 10]. Thus, it seems to be favorable with the current standard treatment modalities (surgery with/without radiation), if we can correctly diagnose the disease entity pathologically. However, among salivary gland cancers, high-grade salivary gland cancers, such as salivary duct carcinomas, have quite different characters in terms of the clinical course and treatment outcomes [11–14]. Aggressive disease progression and early systemic spread are common findings that are observed in these patients [6, 15, 16]. As a result, high-grade salivary gland cancers have one of the highest cancer mortality rates (less than 50% survival in 5 years) among all head and neck cancers [12, 17].

Salivary gland cancers have diverse histopathological subtypes which leads that most of the previous studies have focused on the clinical analyses depending on the specific subtypes or all pathological diagnoses together [18–20]. However, specific pathological diagnosis could not be achieved before detailed examination of the surgical specimen, and preoperative tests are usually insufficient for diagnosing specific pathological subtypes. Thus, overall tumor grade might be a more clinically relevant indicator with respect to treatment decision and prognosis estimation, and it can be diagnosed preoperatively even by fine needle aspiration cytology or core needle biopsy [7, 21, 22].

In line with these assumptions, we evaluated the clinical outcomes of high-grade salivary gland cancers as a whole in this paper. Key question was what could be the important clinical factors that determine the prognosis in patients with high-grade salivary gland cancers. This effort is expected to allow the potential risk stratification of this rare disease with clinical implications.

## Methods

This was a retrospective analysis using clinical data of patients with salivary gland carcinomas, where treatments had followed the National Comprehensive Cancer Network (NCCN) guideline [23]. The study protocol was approved by the Institutional Review Board of Samsung Medical Center, Seoul, Korea. The data used in the study was de-identified.

### Study subjects

The initial study population included 540 patients diagnosed as having salivary gland cancer at our institution from 1995 to 2014. The inclusion criteria for this study were patients who had (i) high-grade salivary gland cancers confirmed by surgical pathology; (ii) no previous treatments for high-grade salivary gland cancer; (iii) more than two years of follow-up from the end of definitive treatment. Patients diagnosed as having metastatic tumors to the salivary gland from other malignancies or recurrent tumors, and those with a previous history of head and neck cancer or irradiation to the head and neck area were excluded. Based on these criteria, clinical data of 139 patients was collected in this study, but 15 patients were further excluded because of incomplete clinical and pathological information, leaving a final *n* = 124 available for the analysis.

Most of the patients (*n* = 103) had initially undergone curative surgery for primary tumors with or without neck dissection. Based on the surgical pathology reports, each tumor was reassigned a pathological tumor-node-metastasis (pTNM) stage using the 7th edition the American Joint Committee on Cancer staging manual [24]. Core needle biopsy or surgical biopsy was conducted to make a pathological diagnosis in the remaining patients (*n* = 21), who had received non-surgical management options, where cT/cN was used in cases instead of pT/pN.

### Pathological diagnosis

To confirm the diagnosis of high-grade salivary gland cancer, a senior pathologist (YHK) with over 10 years’ experience in pathological diagnosis of salivary gland tumors reviewed the surgical specimens. Pathological features, such as extra-parenchymal (extra-glandular) extension, perineural/nerve invasion, lymphovascular invasion/tumor emboli, and resection margin status were redefined in each specimen. In cases of node-positive disease, the number of metastatic nodes and the presence of extracapsular spread were also recorded.

High-grade salivary gland cancer included the following pathology subtypes; salivary duct carcinoma, primary squamous cell carcinoma, solid type adenoid cystic carcinoma [25, 26], high-grade mucoepidermoid carcinoma [18, 27], high-grade adenocarcinoma [28], high-grade carcino-sarcoma and poorly differentiated carcinoma. They were diagnosed based on the histo-morphologic pattern and cytologic features, and only those tumors showing high-grade histology were included. If needed, several immunohistochemical stains were performed to differentiate the pathological subtypes. In cases of squamous cell carcinomas of the salivary glands, we employed all available diagnostic modalities to exclude the possibility of metastasis to the intra-glandular lymph nodes and confirmed the diagnosis of primary squamous cell carcinomas arising from the salivary gland with clinical follow-ups. Equivocal pathological diagnoses were discussed during intra-departmental consultation, and some patients were diagnosed by external review.

### Statistical analyses

In our cohort, we evaluated recurrence and death event according to the treatment modalities, T/N classification and pathological characteristics. pT/pN (or cT/cN in patients with non-surgical management) was used to classify tumor extent. Baseline variables at diagnosis of high-grade salivary gland cancers (age, gender, and primary site) were also considered as the variables for predicting the outcome.

The primary endpoints were disease-specific survival (DSS) in all patients and recurrence-free survival (RFS) in patients with resectable high-grade salivary gland cancers. DSS, RFS and overall survival (OS) were calculated as the time elapsed from the end of definitive treatments until the time of recurrence and death, respectively. The patients without any events (recurrence or death) at the last clinical follow-up were censored. Survival curves were estimated using the Kaplan–Meier method, and group differences were tested using the log-rank test. Prognostic significance of variables was assessed by univariate and multivariate analyses using the Cox proportional hazard model. Statistical analyses were performed using SPSS version 20.0 (IBM Corporation, Armonk, NY, USA). All tests were two-sided and *P* < 0.05 indicated statistical significance.

## Results

### Characteristics of the study subjects

We analyzed the clinical and pathological data of 124 patients with pathologically proven high-grade salivary gland cancer (Table 1). The number of male patients was three times higher than that of female patients (M:F = 3:1) with a mean age of 61 years (range 31 to 89 years). Approximately two-thirds of high-grade salivary gland cancers were found in the parotid gland in our series.

**Table 1:** Characteristics of subjects with total high-grade salivary gland cancers (*n* = 124) and resectable high-grade salivary gland cancers (*n* = 103)

Characteristics	No.	%
Total high-grade salivary gland cancers (n = 124)		
Age [years; median (range)]	61 (31–89)	
Gender (Male/Female)	95/29	76.6/23.4
Tumor site		
Parotid gland	84	67.7
Submandibular gland	38	30.6
Sublingual gland and minor salivary gland	2	1.6
T classification		
T1	20	16.1
T2	44	35.5
T3	23	18.5
T4	37	29.8
N classification		
N0	63	50.8
N1	10	8.1
N2–3	51	41.1
M classification		
M0	109	87.9
M1	15	12.1
AJCC TNM stage		
I	14	11.3
II	24	19.4
III	16	12.9
IV	70	56.5
Pathological diagnosis		
Salivary duct carcinoma	74	59.7
Squamous cell carcinoma, primary*	13	10.5
Adenoid cystic carcinoma, solid type	12	9.7
Mucoepidermoid carcinoma, high-grade	9	7.3
Adenocarcinoma, high-grade	6	4.8
Atypical high-grade carcinoma	3	2.4
Carcino-sarcoma, high-grade	4	3.2
Poorly differentiated carcinoma	3	2.4
Treatment modalities		
Surgery alone	13	10.5
Surgery + adjuvant radiation	62	50.0
Surgery + adjuvant radiation + chemotherapy	28	22.6
Initial non-surgical local treatment (radiation or chemoradiation)	3	2.4
Chemotherapy or palliative treatment	18	14.5
Clinical outcomes		
Disease-specific death	39	31.5
Event-free follow-up period [months; median (range)]	109 [2–188]	
All-cause death	44	35.5
Resectable high-grade salivary gland caners (n = 103)		
Surgery for primary tumor		
R0 resection (cancer cells absent at the resection margin)	97	94.2
R1 resection (cancer cells present at the resection margin)	6	5.8
Neck dissection		
No	31	30.1
Selective neck lymph node dissection	30	29.1
Comprehensive neck lymph node dissection	42	40.8
Pathological risk factors		
Perineural invasion (Y/N)	13/90	12.6/87.4
Lymphovascular invasion (Y/N)	16/87	15.5/84.5
Extra-parenchymal (Extra-glandular) extension of tumor (Y/N)	45/58	43.7/56.3
Extra-capsular spread of lymph node metastasis (Y/N)	26/77	25.2/74.8
Clinical outcomes		
Recurrence	40	38.8
Recurrence-free period [months; median (range)]	109 [2–188]	
Disease-specific death	27	26.2
Event-free follow-up period [months; median (range)]	123 [2–188]	

*Abbreviation*:

AJCC TNM stage: 7th edition of the American Joint Committee on Cancer staging manuals (2010)

Y: presence, N: absence

*No evidence of squamous cell carcinomas in other sites, in imaging studies and clinical follow-ups.

Primary tumor extent was distributed evenly across T classification with 51.6% of T1–2 and 48.3% of T3–4. Similarly, half of high-grade salivary gland cancers showed regional lymph node metastasis (49.2%) at diagnosis. Further, 15 patients (12.1%) already had distant metastasis to the lung (*n* = 12), and lung plus bone (*n* = 3). Among the patients without clinical evidence of systemic spread of disease (M0) (*n* = 109), six patients could not receive the initial surgical treatment because of poor medical condition (*n* = 4) and refusal of surgery (*n* = 2). Thus, 21 patients had received initial non-surgical local treatment or chemotherapy, and 103 subjects had undergone curative surgery for high-grade salivary gland cancers. During clinical courses, we found 39 disease-specific deaths (31.5%) and 44 all-cause deaths (35.5%).

In all patients, the pathological diagnosis was made by surgical pathology or biopsy. Notably, salivary duct carcinoma, excluding the low-grade, non-invasive form of salivary duct carcinoma, comprised 59.7% of high-grade salivary gland cancers, followed by primary squamous cell carcinoma, solid type adenoid cystic carcinoma and high-grade mucoepidermoid carcinoma (7.3–10.5%).

Next, we analyzed the clinical data of patients that underwent an initial curative surgery (*n* = 103). Cervical lymph node dissection was performed in 70% of the patients, however it was not performed in the remaining 30% of the patients, mainly because of small tumor burden with low suspicion of high-grade pathology on pre-operative work-ups, even though these were also proved to be high-grade salivary gland cancer on the final surgical pathology. In these patients, the status of regional lymph nodes was confirmed by radiological findings or clinical follow-ups. Pathological risk factors were found in 12.6 to 43.7% of the patients. After completion of the recommended treatments, 40 recurrences (4 at the primary sites, 11 in the regional lymph nodes and 25 in the distant organs) were detected and 27 disease-specific deaths occurred.

### Disease-specific and overall survival of patients with high-grade salivary gland cancers

The 5-year DSS and OS rates in patients diagnosed as having high-grade salivary gland cancer were 63.4 and 61.4% respectively (*n* = 124). In addition, patients with systemic disease spread survived only a median period of 20 months with a range from 2 to 109 months (*n* = 15 at diagnosis and *n* = 26 detected during the clinical course). As responsible prognostic factors, lymph node and distant metastases at presentation were identified as the most significant indicators of poor survival (Tables 2 and 3). Intriguingly, specific pathological subtypes of high-grade salivary gland cancers were not major determinants of patient survival, suggesting the need for a clinical approach to high-grade salivary gland cancer as a whole.

**Table 2:** Disease–specific survival in patients with high-grade salivary gland cancers (*n* = 124)

Factors (Number)	Univariate model			Multivariate model #1			Multivariate model #2		
	HR	95% CI	*P*	HR	95% CI	*P*	HR	95% CI	*P*
Age (years)	1.021	0.994–1.049	0.135						
Gender (Male/Female) (95/29)	1.446	0.636–3.290	0.379						
Primary site									
Parotid gland (84)	1 (Ref.)								
Non-parotid gland (40)	1.246	0.640–2.427	0.518						
TNM categories									
T3–4/T1–2 (60/64)	1.880	0.980–3.608	0.058	0.824	0.412–1.648	0.584	1.145	0.593–2.212	0.687
N1–3/N0 (61/63)	5.573	2.651–11.713	< 0.001	8.669	3.787–19.842	< 0.001	5.632	2.638–12.027	< 0.001
M1/M0 (15/109)	4.550	2.139–9.680	< 0.001				4.591	2.100–10.035	< 0.001
Pathological diagnosis									
Salivary duct carcinoma (74)	1 (Ref.)								
Squamous cell carcinoma, primary (13)	1.271	0.480–3.368	0.629						
Adenoid cystic carcinoma, solid type (12)	1.422	0.537–3.766	0.479						
Mucoepidermoid carcinoma, high-grade (9)	1.094	0.326–3.673	0.885						
Adenocarcinoma, high-grade (6)	0.386	0.052–2.870	0.352						
Others^*^ (10)	1.681	0.501–5.642	0.440						
Treatment modalities									
Surgery (13)	1 (Ref)			1 (Ref.)					
Surgery + radiation (62)	1.247	0.372–4.182	0.721	0.761	0.216–2.678	0.671			
Surgery + radiation + chemotherapy (28)	0.418	0.084–2.075	0.286	0.134	0.025–0.709	0.018			
Others^†^ (21)	4.589	1.281–16.434	0.019	2.786	0.731–10.617	0.133			

M1 status was significantly associated with the application of the so-called other treatment modalities (initial non-surgical, chemotherapy or palliative treatments); thus, we built two separate multivariate models using independent variables

*Abbreviation*:

*HR* Hazard ratio, *CI* Confidence interval

Others^*^: Atypical high-grade carcinoma, high-grade carcino-sarcoma, poorly differentiated carcinoma

Others^†^: Initial non-surgical local treatment (radiation or chemoradiation), chemotherapy or palliative treatment

**Table 3:** Overall survival in patients with high-grade salivary gland cancers (*n* = 124)

Factors (Number)	Univariate model			Multivariate model #1			Multivariate model #2		
	HR	95% CI	*P*	HR	95% CI	*P*	HR	95% CI	*P*
Age (years)	1.032	1.005–1.059	0.019	1.038	1.008–1.069	0.012	1.054	1.024–1.084	< 0.001
Gender (Male/Female) (95/29)	1.396	0.645–3.022	0.397						
Primary site									
Parotid gland (84)	1 (Ref.)								
Non-parotid gland (40)	1.286	0.687–2.405	0.432						
TNM categories									
T3–4/T1–2 (60/64)	1.785	0.955–3.336	0.069	0.807	0.405–1.609	0.542	0.964	0.505–1.839	0.912
N1–3/N0 (61/63)	5.002	2.492–10.042	< 0.001	9.263	4.025–21.317	< 0.001	7.010	3.176–15.472	< 0.001
M1/M0 (15/109)	4.171	1.978–8.795	< 0.001				4.716	2.148–10.357	< 0.001
Pathological diagnosis									
Salivary duct carcinoma (74)	1 (Ref.)								
Squamous cell carcinoma, primary (13)	1.434	0.582–3.534	0.433						
Adenoid cystic carcinoma, solid type (12)	1.381	0.523–3.646	0.515						
Mucoepidermoid carcinoma, high-grade (9)	1.458	0.501–4.246	0.489						
Adenocarcinoma, high-grade (6)	0.370	0.050–2.752	0.332						
Others^*^ (10)	2.059	0.707–5.999	0.186						
Treatment modalities									
Surgery (13)	1 (Ref)			1 (Ref.)					
Surgery + radiation (62)	0.945	0.355–2.514	0.909	0.720	0.254–2.044	0.537			
Surgery + radiation + chemotherapy (28)	0.299	0.070–1.276	0.103	0.158	0.033–0.755	0.021			
Others^†^ (21)	3.282	1.115–9.655	0.031	1.793	0.374–8.602	0.466			

M1 status was significantly associated with the application of the so-called other treatment modalities (initial non-surgical, chemotherapy or palliative treatments); thus, we built two separate multivariate models using independent variables

*Abbreviation*:

*HR* hazard ratio, *CI* confidence interval

Others^*^: Atypical high-grade carcinoma, high-grade carcino-sarcoma, poorly differentiated carcinoma

Others†: Initial non-surgical local treatment (radiation or chemoradiation), chemotherapy or palliative treatment

**Fig. 1:**
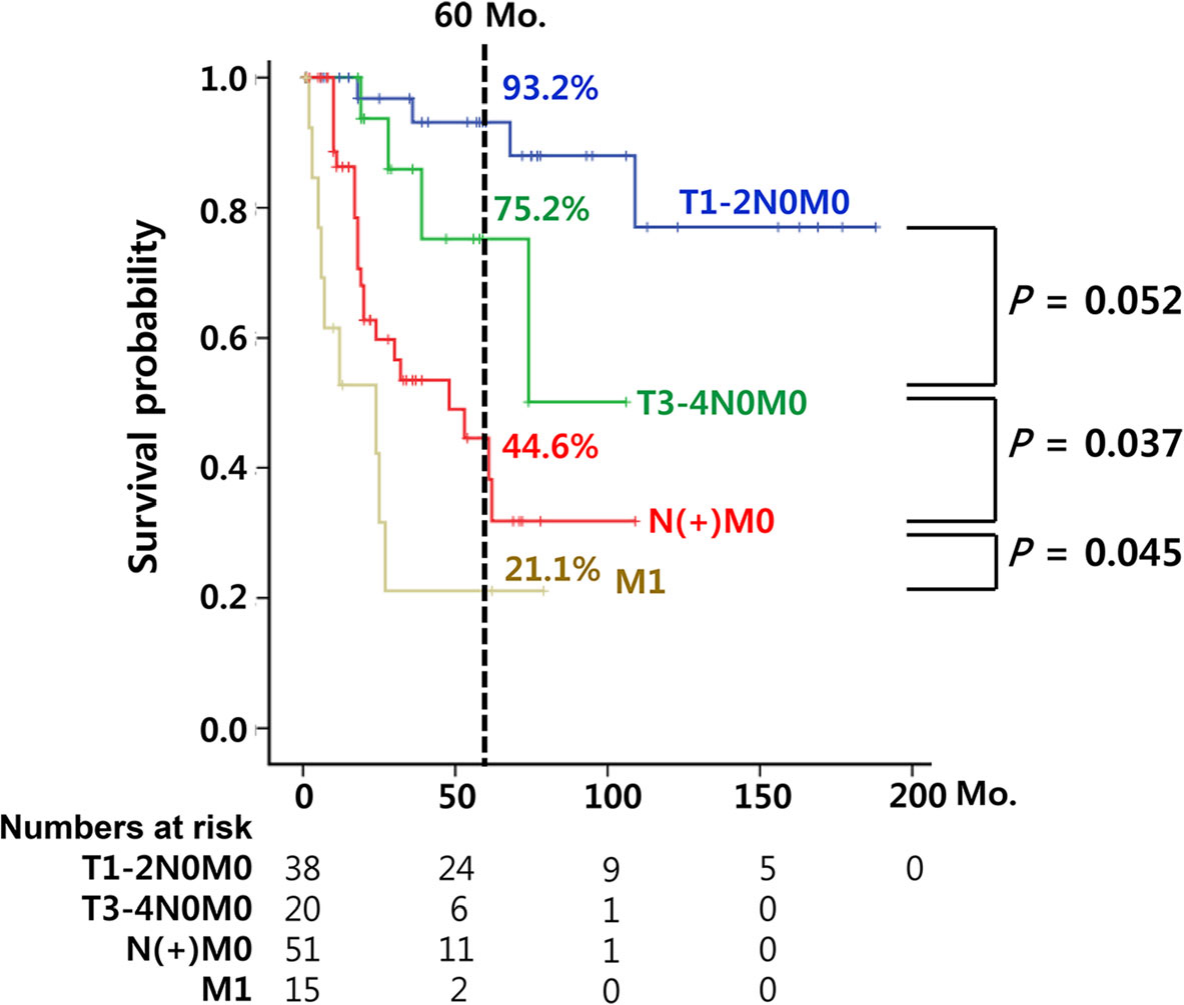
Survival curves according to the tumor-node-metastasis staging in patients with high-grade salivary gland cancers

Next, we performed multivariate survival analyses using the variables with significant values on the univariate analyses. Because M1 status at diagnosis was the main determinant of initial non-surgical treatments (multi-collinearity), we separately built two independent multivariate models to minimize interference. In the analyses, we confirmed that lymph node and distant metastases were significant independent predictors of poor disease-specific and overall survival in patients with high-grade salivary gland cancers. In addition, patient age was another significant factor for overall survival (Table 3). Kaplan-Meier survival analyses also showed clear discrimination of the survival plots between non-metastatic and metastatic high-grade salivary gland cancer (Fig. 1). Of note, early stage high-grade salivary gland cancer showed excellent prognosis (93.2% 5-year DSS), indicating the significance of early diagnosis for improving treatment outcomes.

On comparing surgery alone as a treatment modality, surgery plus postoperative radiation and chemotherapy significantly benefited the patients with high-grade salivary gland cancers, commonly having advanced stage disease (Tables 2 and 3). However, the difference between adjuvant radiation and chemoradiation was not found to be significant. Most of T1–2 diseases were treated with surgery plus radiation, except for a subset of T1 tumors with adequate resection margins on surgery.

### Recurrence-free and disease-specific survival of patients with resectable high-grade salivary gland cancers

**Fig. 2:**
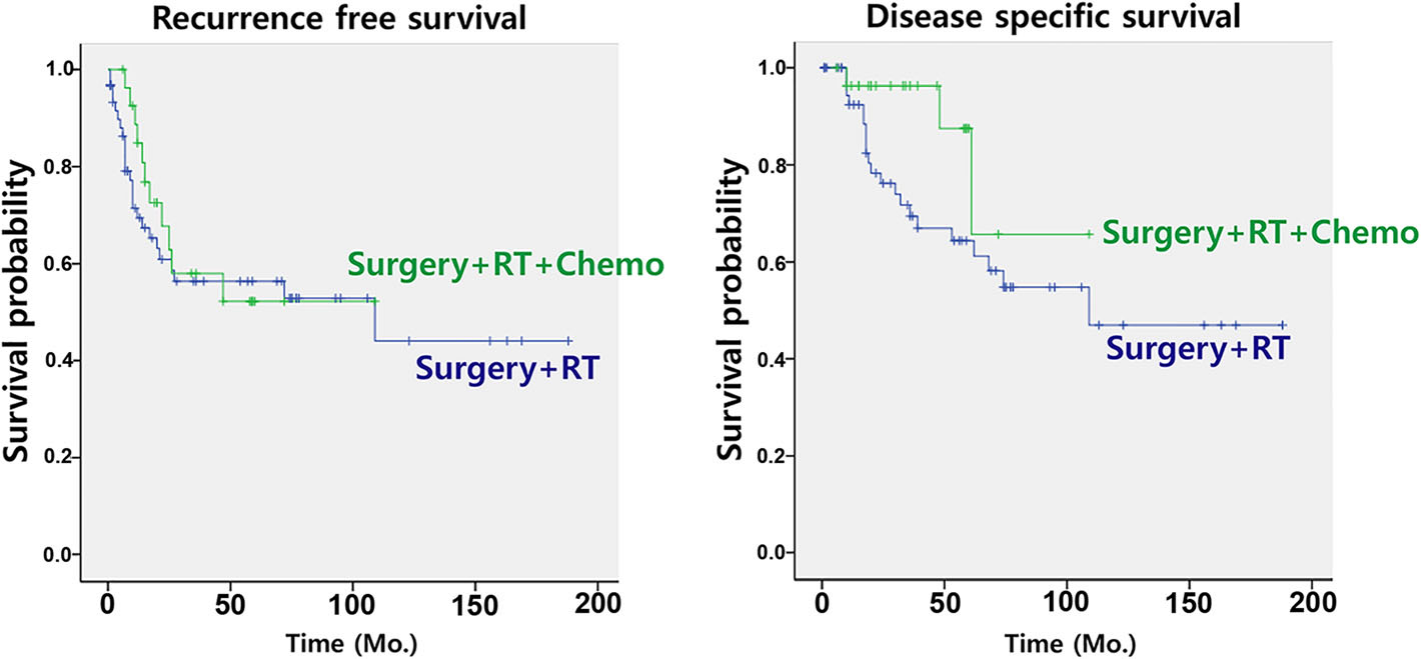
Comparison of survivals between the two treatment strategies: surgery plus post-operative radiation versus surgery plus post-operative radiation and chemotherapy for high-grade salivary gland cancer

Using the patient data with resectable high-grade salivary gland cancer (*n* = 103), we constructed two separate Cox proportional hazard models, because a pathological risk factor, extra-parenchymal extension of the primary tumor was significantly associated with T3 classification (Table 4). Among various clinical and pathological variables, lymph node metastasis was only found to be significant as an independent prognostic factor for poor RFS in patients with resectable high-grade salivary gland cancers, and none of the pathological risk factors showed significance in survival analyses. This result was also consistent with DSS in patients with resectable high-grade salivary gland cancer (Table 5). Similarly, surgery plus postoperative chemoradiation was associated with better DSS than surgery alone. However, no survival difference between adjuvant radiation and chemoradiation was observed, which was same as the results obtained in total patients with high-grade salivary gland cancers (Fig. 2).

**Table 4:** Recurrence-free survival in patients with resectable high-grade salivary gland cancers (*n* = 103)

Factors (Number)	Univariate model			Multivariate model #1			Multivariate model #2		
	HR	95% CI	*P*	HR	95% CI	*P*	HR	95% CI	*P*
Age (years)	1.008	0.980–1.036	0.584						
Gender (Male/Female) (83/20)	2.070	0.808–5.303	0.130						
Primary site									
Parotid gland (69)	1 (Ref.)								
Non-parotid gland (34)	1.718	0.912–3.237	0.094						
TN categories									
T3–4/T1–2 (45/58)	1.986	1.048–3.761	0.035	0.984	0.460–2.103	0.967			
N1–3/N0 (48/55)	4.489	2.241–8.994	< 0.001	4.693	1.990–11.069	< 0.001	4.650	1.967–10.994	< 0.001
Pathological diagnosis									
Salivary duct carcinoma (67)	1 (Ref.)								
Squamous cell carcinoma, primary (9)	0.226	0.031–1.661	0.114						
Adenoid cystic carcinoma, solid type (8)	1.289	0.451–3.685	0.635						
Mucoepidermoid carcinoma, high-grade (7)	0.851	0.258–2.806	0.791						
Adenocarcinoma, high-grade (6)	0.283	0.040–2.162	0.229						
Others (6)	1.847	0.556–6.138	0.317						
Treatment modalities									
Surgery (13)	1 (Ref)			1 (Ref)			1 (Ref)		
Surgery + radiation (62)	1.294	0.450–3.719	0.632	0.917	0.303–2.778	0.879	0.912	0.301–2.758	0.870
Surgery + radiation + chemotherapy (28)	1.068	0.339–3.359	0.911	0.421	0.125–1.425	0.164	0.421	0.125–1.424	0.164
Surgery of primary tumor (R1/R0) (6/97)	1.126	0.347–3.660	0.843						
Neck dissection/no neck dissection (72/31)	1.489	0.739–3.000	0.266						
Pathological risk factors									
Perineural invasion (Y/N) (13/90)	1.111	0.434–2.846	0.827						
Lymphovascular invasion (Y/N) (16/87)	1.132	0.440–2.915	0.797						
Extra-parenchymal extension (Y/N) (45/58)	2.045	1.077–3.886	0.029				1.006	0.469–2.157	0.988
Extra-capsular spread (Y/N) (26/77)	3.236	1.665–6.290	0.001	1.799	0.850–3.811	0.125	1.792	0.846–3.795	0.128

T classification (particularly T3) was significantly associated with the presence of extra-parenchymal extension of primary tumors; thus, we built two separate multivariate models using independent variables

*Abbreviation*:

*HR* hazard ratio *CI* confidence interval

R1 resection: Cancer cells present at the resection margin, R0 resection: Cancer cells absent at the resection margin

Others (Pathology diagnosis): Atypical high-grade carcinoma, high-grade carcino-sarcoma, poorly differentiated carcinoma

Y: presence, N: absence

**Table 5:** Disease-specific survival in patients with resectable high-grade salivary gland cancers (*n* = 103)

	Univariate model			Multivariate model		
	HR	95% CI	*P*	HR	95% CI	*P*
Age (years)	1.012	0.980–1.045	0.464			
Gender (Male/Female) (83/20)	2.325	0.697–7.756	0.170			
Primary site						
Parotid gland (69)	1 (Ref.)					
Non-parotid gland (34)	1.409	0.644–3.081	0.390			
TN categories						
T3–4/T1–2 (45/58)	2.011	0.919–4.403	0.080	0.744	0.285–1.938	0.544
N1–3/N0 (48/55)	6.115	2.509–14.903	< 0.001	10.211	3.485–29.919	< 0.001
Pathological diagnosis						
Salivary duct carcinoma (67)	1 (Ref.)					
Squamous cell carcinoma, primary (9)	0.627	0.145–2.718	0.533			
Adenoid cystic carcinoma, solid type (8)	1.557	0.457–5.308	0.479			
Mucoepidermoid carcinoma, high-grade (7)	0.541	0.072–4.069	0.550			
Adenocarcinoma, high-grade (6)	0.439	0.058–3.302	0.424			
Others (6)	1.978	0.455–8.604	0.363			
Treatment modalities						
Surgery (13)	1 (Ref)			1 (Ref)		
Surgery + radiation (62)	1.266	0.377–4.248	0.702	0.742	0.207–2.655	0.646
Surgery + radiation + chemotherapy (28)	0.433	0.087–2.155	0.307	0.121	0.022–0.677	0.016
Surgery for primary tumor (R1/R0) (6/97)	0.045	0.000–39.169	0.369			
Neck dissection/no neck dissection (72/31)	1.871	0.780–4.488	0.161			
Pathological risk factors						
Perineural invasion (Y/N) (13/90)	0.042	0.000–12.297	0.274			
Lymphovascular invasion (Y/N) (16/87)	0.043	0.000–20.431	0.317			
Extra-parenchymal extension (Y/N) (45/58)	2.156	0.979–4.750	0.057			
Extra-capsular spread (Y/N) (26/77)	2.547	1.065–6.092	0.036	1.363	0.509–3.649	0.538

*Abbreviation*:

*HR* hazard ratio, *CI* confidence interval

R1 resection: Cancer cells present at the resection margin, R0 resection: Cancer cells absent at the resection margin

Others (Pathological diagnosis): Atypical high-grade carcinoma, high-grade carcino-sarcoma, poorly differentiated carcinoma

Y: presence, N: absence

## Discussion

High-grade salivary gland cancer is a rare disease entity; however it causes fatal cancer-related consequences in most of the patients. Unfortunately, clinical and basic research studies are limited because of the rarity of the disease, and most of the clinical information on the optimal treatments has been obtained from the retrospective studies.

In our series, the 5-year disease-specific survival in patients with lymph node metastasis was 44.6% while the disease-specific survival was 63.4% in all patients with high-grade salivary gland cancers. These results were concordant with the previous studies reporting the estimated survival of 30.6–44.0% in patients with N (+) salivary gland cancers while reporting the estimated survival of 64.3–80.0% in patients with non-metastatic salivary gland cancers [12, 13, 29, 30]. Our data also showed that the patients with systemic disease spread survived only a median period of 20 months, which is concordant with the previous reports indicating the median survival of 15 months after distant metastasis development [15]. To sum up our findings and previous reports, the presence of metastasis to regional lymph node or distant organ is a significant independent prognostic factor for survival in patients with high-grade salivary gland cancers. Meanwhile, high-grade salivary gland cancer that did not have metastasis at presentation showed a relatively favorable outcome (93.2% in T1–2N0M0 and 75.2% in T3–4N0M0, Fig. 1), even in high-grade pathology while performing surgery plus adjuvant radiation in most patients. Thus, this suggests that early detection or diagnosis of high-grade salivary gland cancer is very important for improving patients’ prognosis, before high-grade salivary gland cancer progresses to clinically overt metastasis.

**Fig. 3:**
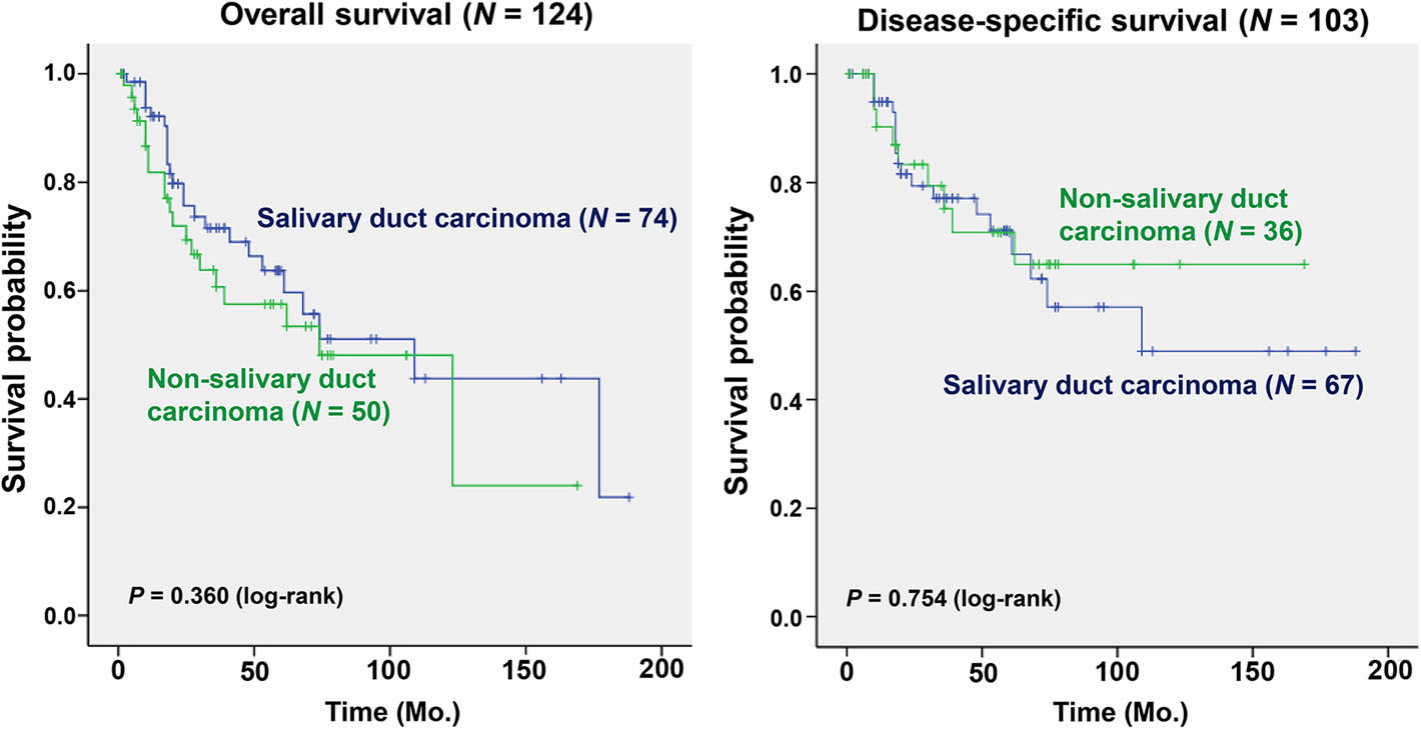
Comparison of survivals in patients diagnosed with salivary duct carcinomas and non-salivary duct carcinoma pathologies

**Fig. 4:**
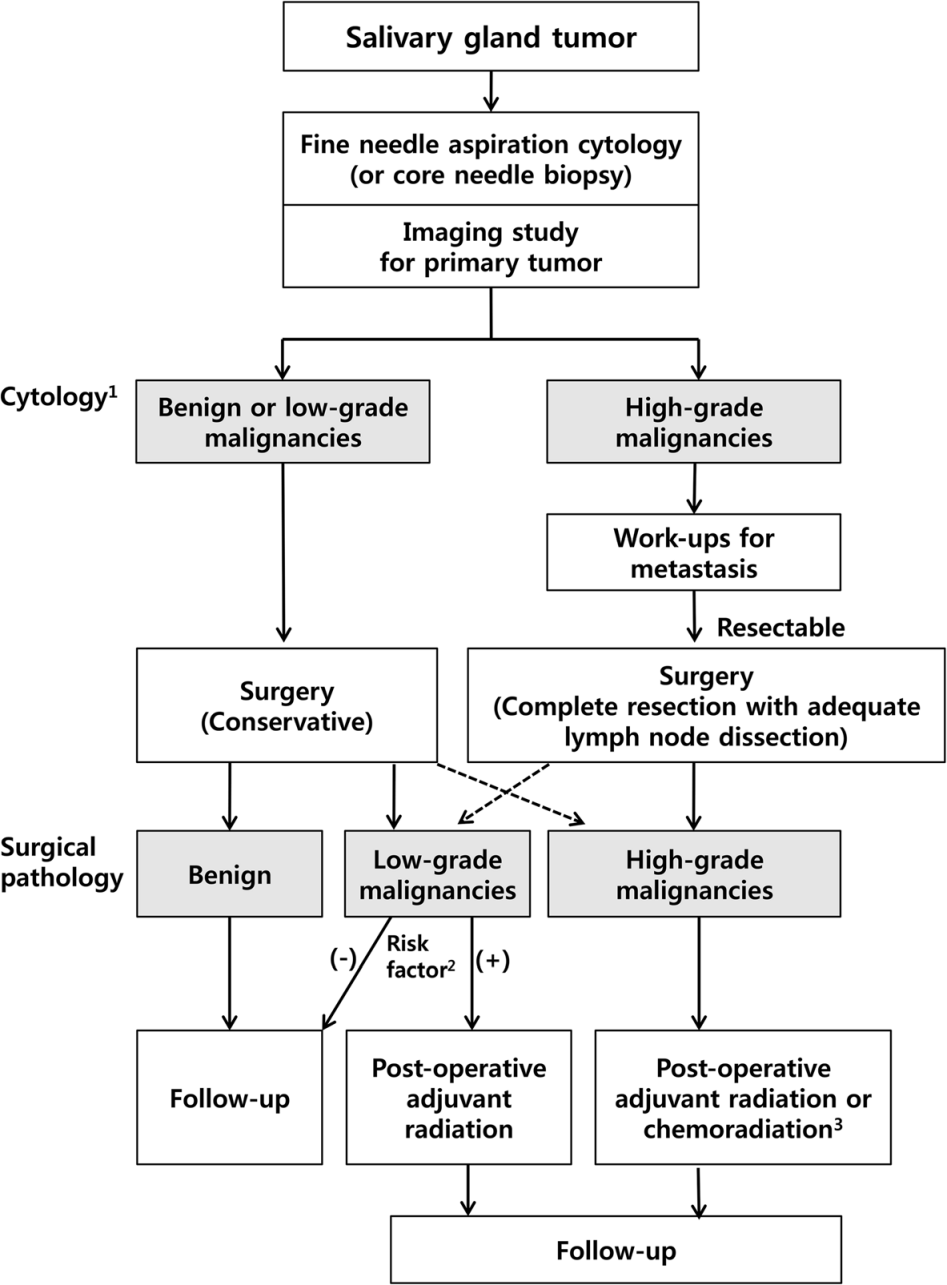
Tumor grade-based management strategy for salivary gland tumors ^1^Cytology: Reference [7], ^2^Risk factors: Reference [9], ^3^chemoradiation: requires further clinical validation

Another interesting point in our study was that there was no significant difference among high-grade subtypes in terms of treatment outcomes and patient survival. High-grade salivary duct carcinoma, which is already known to have a dismal outcome, was the most frequent subtype of high-grade salivary gland cancer in our series, but other pathologic types of high-grade salivary gland cancers also had a similar clinical course (Fig. 3). Solid subtype of adenoid cystic carcinoma is a distinct form, different from the cribriform or tubular subtypes, which also has frequent lymph node and distant metastases, with a relatively rapid disease progression [26]. Therefore, our findings and the previous reported results suggest a clinical approach to salivary gland tumor suspicious of high-grade salivary gland cancer as a whole [6]. These results provide a clinical implication on the management strategy of salivary gland neoplasm that pre-operative workup might focus on the discrimination of tumor grade rather than the discrimination of specific pathological subtype (Fig. 4).

Frequently, current pre-operative diagnostic work-ups could not differentiate the specific pathological subtypes of high-grade salivary gland cancer with an enough diagnostic accuracy [7]. However, molecular or genetic testing to understand tumor biology and estimated prognosis are now developing [31, 32], which could be helpful even in the pre-operative settings. Previously, we reported that fine needle aspiration cytology could detect high-grade tumors with an acceptable diagnostic accuracy (90%), although it has difficulty in discriminating benign versus malignant disease [7, 22]. Core needle biopsy may also be beneficial to differentiate high-grade pathology. Thus, fine needle aspiration cytology or core needle biopsy with appropriate radiological work-up can be sufficient for differential diagnoses of benign/low-grade malignancies and high-grade malignancies in pre-treatment evaluation and treatment decision for salivary gland tumor (Fig. 4).

With respect to the salivary lesions suspicious for high-grade salivary gland cancer, pre-treatment workup for metastasis seems mandatory, because in our case series, approximately 50% of high-grade salivary gland cancers had lymph node metastasis at diagnosis, and 12% of high-grade salivary gland cancers had distant metastasis. A recent study also showed that 56% of patients had lymph node metastasis at diagnosis even in early T stage salivary duct carcinoma [33]. Initial surgery for resectable high-grade salivary gland cancers should include the complete removal of primary tumors and the potential nodal metastasis. In addition, adjuvant radiation with/without chemotherapy is recommended for high-grade salivary gland cancers [23]. In fact, most patients of early stage high-grade salivary gland cancers in our study had received adjuvant radiation because previous evidence already showed that the adjuvant radiation in salivary gland cancer with risk factors including high grade pathology increased the patient survivals [34, 35]. However, the role of adjuvant chemoradiation is still controversial [34] and under Phase 3 clinical trials (ClinicalTrials.gov Identifier: NCT01220583). Collectively, we suggest a clinical management strategy for high-grade salivary gland cancer, which includes tumor grade-based diagnostic work-ups and management, surgery with adjuvant radiation and/or chemotherapy for high-grade or low-grade salivary gland cancer with risk factors (Fig. 4).

To gain better treatment outcomes in high-grade salivary gland cancers, we cautiously suggest a screening program (self-palpation or imaging) for patients susceptible to salivary gland tumor (e.g. family history, susceptible age), such as many other solid cancers. Indeed, as demonstrated previously (Fig. 1), early detection of high-grade salivary gland cancer before the occurrence of clinical metastasis appears to be the best option for improving outcomes of these patients, because patients diagnosed as having high-grade salivary gland cancer without metastasis have a high chance of cure from these devastating diseases. Majorities of salivary gland cancer arise from the parotid gland and submandibular gland which are easily palpable, thus education program of self-palpation might be effective for detecting the neoplasm in early-stage.

This study had several limitations, which the readers should keep in mind while interpreting our results. As mentioned earlier, the study collected clinical data retrospectively; therefore we could not compare the survival benefit of each treatment modality without selection bias. Therefore, superiority and benefit of postoperative adjuvant multimodal treatment should be re-evaluated in future prospective studies. In addition, we included a relatively large number of high-grade salivary gland cancer patients from a total of 540 patients with salivary gland cancers into our analyses; however the number of subjects was still not enough because some subtypes of high-grade salivary gland cancers are very rare. Thus, it was possible that our combined results did not reflect the unique features of rare subtypes of high-grade salivary gland cancers. Nevertheless, we think that our clinical approach based on high-grade pathology seems to be clinically practical, because of disease rarity and pathological diversity. Recently, a similar simplified, but combined approach to adenoid cystic carcinoma has been proposed, suggesting the differentiation of solid versus non-solid components [26].

## Conclusions

Our data demonstrated that the presence of metastasis (nodal or distant) was the most significant prognostic factor for worse survival among patients with high-grade salivary gland cancers. Considering that the prognosis of early stage high-grade salivary gland cancer was relatively favorable, a public screening program, for example a self-palpation or education for general population might be helpful to detect high-grade salivary gland cancer in early-stage.

## Abbreviations

DFS: disease-specific survival
OS: overall survival.
RFS: recurrence-free survival
TNM staging: Tumor-node-metastasis staging
